# Comparison of Survival Outcomes of Breast-Conserving Surgery Plus Radiotherapy with Mastectomy in Early Breast Cancer Patients: Less Is More?

**DOI:** 10.3390/cancers17040591

**Published:** 2025-02-09

**Authors:** Chularat Duangkaew, Areewan Somwangprasert, Kirati Watcharachan, Phanchaporn Wongmaneerung, Wasana Ko-iam, Issara Kaweewan, Chagkrit Ditsatham

**Affiliations:** 1 Division of Head, Neck, and Breast Surgery, Department of Surgery, Faculty of Medicine, Chiang Mai University, 110 Intavaroros Road, Amphoe Muang, Chiang Mai 50200, Thailand; chularat.d@cmu.ac.th (C.D.);; 2 Clinical Surgical Research Center, Faculty of Medicine, Chiang Mai University, Chiang Mai 50200, Thailand; 3 Research Unit, Department of Surgery, Faculty of Medicine, Chiang Mai University, Chiang Mai 50200, Thailand; 4 Faculty of Medicine, Chiang Mai University, Chiang Mai 50200, Thailand

**Keywords:** breast-conserving therapy, mastectomy, survival outcome, survival, early breast cancer

## Abstract

Purpose: To compare the survival outcomes of early-stage breast cancer patients treated with breast-conserving therapy (BCT) and mastectomy. Method: This retrospective study includes 1330 early-stage breast cancer patients treated at Chiang Mai University (CMU) hospital, using data from the Chiang Mai Cancer Registry between 2004 and 2015. Information pertinent to patients and their treatment was collected for analysis. Time-to-event analysis was performed using Kaplan–Meier methods. Results: The baseline characteristics of 1330 patients showed significant differences between the BCT and mastectomy groups in terms of age, tumor size, and tumor location. BCT patients were younger, had smaller tumors, and exhibited less nodal involvement. Propensity score matching created a balanced cohort of 534 patients where differences persisted in age and tumor size. Univariate analysis revealed significant survival associations for BCT, younger age, and smaller tumor size. Multivariate analysis confirmed these factors, with BCT showing an adjusted hazard ratio (HR) of 0.58 (95% CI: 0.36–0.93; *p* = 0.023) compared to mastectomy. Kaplan–Meier survival analysis demonstrated a significant survival advantage for BCT, particularly in HER2-enriched and triple-negative subtypes. The 15-year overall survival was 80.01% in the BCT group versus 64.33% in the mastectomy group (*p* < 0.001). Conclusions: This study reveals key differences between outcomes following breast-conserving therapy (BCT) and mastectomy patients, including age and tumor characteristics. BCT showed improved overall survival, particularly in HER2-enriched and triple-negative breast cancers. However, our study’s limitations may affect the results. These findings suggest that BCT may offer survival benefits for specific subtypes, highlighting the importance of personalized treatment approaches.

## Simple Summary

Surgery remains the primary treatment for early-stage breast cancer. Several clinical trials have been conducted to examine the comparative effectiveness and oncologic safety of breast-conserving therapy (BCT) and mastectomy. Recent studies suggest that BCT may be associated with improved survival rates compared to mastectomy. This retrospective study aims to compare the survival outcomes of early-stage breast cancer patients treated with these two approaches. Our findings reveal that BCT improved overall survival, particularly in HER2-enriched and triple-negative subtypes. These results indicate that BCT may offer survival advantages for certain subtypes, underlining the need for personalized treatment approaches.

## 1. Introduction

Breast cancer is the most prevalent type of cancer among women worldwide and represents a significant global health concern [1,2]. It is a leading cause of death in women, with the incidence of breast cancer rising, particularly in developing countries. In Thailand, breast cancer remains the most common cancer among women, and its prevalence continues to rise [3]. This increase can be attributed to improved screening methods and greater accessibility to healthcare for early-stage breast cancer (EBC).

Understanding the interplay between breast cancer subtypes and their clinical and pathological stages is critical for optimizing treatment strategies. Ejlertsen [4] emphasized the importance of molecular subtyping in guiding the use of chemotherapy, endocrine therapy, and radiotherapy in early breast cancer. Similarly, Choi et al. [5] demonstrated how the TNM staging system can be effectively adapted to breast cancer subtypes to inform tailored adjuvant treatment strategies. Mustacchi et al. [6] provided detailed criteria for selecting adjuvant chemotherapy regimens based on the biological characteristics of different subtypes, underlining the heterogeneity in therapeutic responses. Van Maaren et al. [7] focused on the clinical significance of achieving a pathologic complete response in early breast cancer, revealing that this outcome varies significantly by subtype and is a predictor of improved survival. Speers et al. [8] examined the role of molecular profiling in guiding postoperative radiotherapy decisions, showcasing its impact on improving outcomes in breast-conserving therapy for early-stage breast cancer. Langlands et al. [9] analyzed the differential responses of molecular subtypes to radiotherapy, emphasizing the potential for subtype-specific radiosensitization strategies. The unique challenges and treatment outcomes associated with triple-negative breast cancer were addressed by Wang et al. [10]. They demonstrated the benefits of combining adjuvant chemotherapy and radiotherapy for this aggressive subtype.

The 5-year relative survival rate for EBC is estimated to be as high as 99%, according to the Surveillance, Epidemiology, and End Result (SEER) database [11]. This has led to a trend towards de-escalating treatment for EBC; however, surgical procedures remain the cornerstone of treatment. Mastectomy has long been regarded as the mainstay approach for EBC. Sir Geoffrey Keynes, an English surgeon at St. Bartholomew’s Hospital in London, first described breast-conservation surgery (BCS) for breast cancer in 1924 [12]. Following this initial description, trials were conducted to determine whether BCS was equivalent to mastectomy in terms of effectiveness [13,14,15,16,17].

Several studies have compared the outcomes of BCS combined with radiotherapy (RT) to those associated with mastectomy, finding that breast-conserving therapy (BCT) or BCS plus RT may be associated with improved survival in breast cancer [18,19,20,21,22]. Meta-analysis demonstrated that mastectomy had a slight overall survival (OS) advantage compared to BCT in EBC, albeit with a minimal benefit [23,24]. Conversely, a recent large meta-analysis showed better OS for BCT compared to mastectomy [25]. This discrepancy highlights the ongoing debate in the field.

The aim of this study was to compare the long-term survival outcomes of BCT with those of mastectomy in women with EBC.

## 2. Materials and Methods

This is a retrospective cohort observational study that compares survival among breast cancer patients treated at Chiang Mai University (CMU) Hospital, utilizing data from the Chiang Mai Cancer Registry from 2004 to 2015. This study included women aged 18 to 80 who were diagnosed with invasive breast cancer and underwent surgical treatment, specifically those with pathologic stages T1-2 and N0-1. Patients diagnosed with locally advanced breast cancer, stage IV breast cancer or metastatic disease, inflammatory breast cancer, Paget’s disease of the nipple, or those with incomplete data were excluded from this study. The primary endpoint was overall survival (OS), defined as the time from the surgery until death from any cause.

### 2.1. Statistics

Descriptive statistics for categorical variables were calculated using frequency and percentages, while continuous variables with a normal distribution were analyzed using mean values and standard deviations. For continuous data that did not follow a normal distribution, medians and interquartile ranges were employed. When data were normally distributed, independent or dependent samples were analyzed using *t*-tests. For nonnormally distributed data, the Mann–Whitney U test served as a nonparametric alternative. To compare proportions between different groups within categorical data, a chi-squared test was utilized. Propensity score matching was applied to estimate the effect of treatment on survival outcome. Time-to-event analysis was conducted using Kaplan–Meier methods, and the log-rank test was employed to access the association between each prognostic factor and overall survival (OS). Statistical analyses were performed using Stata version 16 (StataCorp, College Station, TX, USA). *p* < 0.05 was considered statistically significant.

### 2.2. Data Collection

Patient information such as age at diagnosis, medical insurance, marital status, and menopausal status were collected from electronic medical records and pathological reports, in addition to details pertinent to the tumor such as the size, location, stage, type, grade, number of positive lymph nodes, pathological stage, Estrogen receptor(ER) and Progesterone receptor(PR) status, and Human Epidermal growth factor Receptor 2(HER2) status. Tumor positivity for ER or PR was defined as 1% or more of tumor cells showing nuclear staining, and a positive HER2 result was indicated by a score of 2+ on immunohistochemistry (IHC) testing and required confirmation using either fluorescent in situ hybridization (FISH) or a score of 3+ on IHC testing. Treatment information, including the surgical procedure, use of adjuvant chemotherapy, hormonal therapy, and radiotherapy, was also collected.

## 3. Results

### 3.1. Patient Demographics and Clinical Characteristics

Table 1 presents the baseline characteristics of the breast cancer patients in this study. A total of 1330 patients underwent either breast-conserving therapy (BCT) or mastectomy before propensity score matching, with 293 patients in the BCT group and 1037 in the mastectomy group. The mean age of patients in the BCT group was substantially younger, at 47.88 years (SD 10.17), compared to 52.71 years (SD 10.70) in the mastectomy group (*p* < 0.001). This difference was further emphasized by the age distribution: 59.18% of BCT patients were under 50, compared to 40.45% in the mastectomy group.

Menopausal status showed a marked difference. A higher percentage of BCT patients were premenopausal (54.88%) compared to 37.91% in the mastectomy group (*p* < 0.001). The location of tumors showed significant variation (*p* = 0.001). In the BCT group, the upper outer quadrant was the most common tumor site (45.62%), followed by the upper inner quadrant (30.41%). In the mastectomy group, the distribution was more diverse, with 44.19% of tumors located in the upper outer quadrant, followed by 20.27% in the upper inner quadrant and a higher proportion in the central quadrant (12.70% vs. 5.53% in the BCT group). Tumor size was significantly smaller in the BCT group, with a mean size of 1.73 cm (SD 0.94) compared to 2.16 cm (SD 1.15) in the mastectomy group (*p* < 0.001). A higher proportion of BCT patients had tumors measuring 0–2 cm (69.62% vs. 51.78%), whereas tumors measuring 2.1–5 cm were more frequent in the mastectomy group (48.22% vs. 30.38%). Nodal staging did not show statistically significant differences; most patients in both groups were N0 (95.22% in BCT vs. 91.80% in mastectomy; *p* = 0.058).

Histological type was predominantly ductal carcinoma in both groups, with no significant difference observed (93.10% in BCT vs. 93.70% in mastectomy; *p* = 0.686). Tumor grade distribution was also similar between the two groups. No significant differences were noted in hormonal receptor status. Positive estrogen receptor (ER) status and progesterone receptor status (PR) were similar between the two groups. No significant differences were noted in HER2 receptor expression (*p* = 0.094). Molecular subtype analysis revealed differences between the groups (*p* = 0.047). The BCT group had a higher proportion of Luminal B tumors (39.27% vs. 33.05% in mastectomy), while HER2-enriched tumors were more common in mastectomy patients (34.91% vs. 40.97%). Triple-negative tumors were more frequent in the BCT group (18.91% vs. 15.84%).

Adjuvant chemotherapy was similarly utilized in both groups, with 75.70% of BCT patients and 75.47% of mastectomy patients receiving this therapy, showing no statistically significant difference (*p* = 1.000). Likewise, adjuvant hormonal therapy was administered to 61.29% of BCT patients and 66.70% of mastectomy patients, with the difference also being nonsignificant (*p* = 0.101). In contrast, the use of adjuvant radiotherapy demonstrated a substantial disparity. Among BCT patients, 79.72% received radiotherapy, compared to only 34.87% of mastectomy patients (*p* < 0.001). This difference reflects the standard clinical practice where radiotherapy is routinely combined with BCS to ensure local control, whereas it is selectively used following mastectomy based on risk factors. The use of adjuvant targeted therapy, involving agents such as trastuzumab, was low in both groups and did not differ significantly. Only 5.67% of BCT patients and 5.29% of mastectomy patients received targeted therapy (*p* = 0.767).

The analysis of Table 2 presents the characteristics of 534 patients (267 in each group) following propensity score matching, comparing those treated with breast-conserving therapy (BCT) and mastectomy. The mean age of patients in the BCT group was significantly younger, at 47.60 years (SD 10.14), compared to 52.27 years (SD 10.78) in the mastectomy group (*p* < 0.001). When categorized, 59.18% of BCT patients were younger than 50 years, whereas only 40.45% of mastectomy patients fell into this age group (*p* < 0.001). Tumor size was significantly different between the two groups. Tumors measuring 0–2 cm were more prevalent in the BCT group (68.91%) compared to the mastectomy group (49.44%). Conversely, tumors measuring 2.1–5 cm were more frequent in the mastectomy group (50.56%) compared to the BCT group (31.09%; *p* < 0.001). The distribution of tumor grades was comparable between the two groups. Nodal staging showed a nonsignificant difference between the groups. In the BCT group, 95.13% of patients were classified as N0 compared to 91.01% in the mastectomy group. N1 involvement was more frequent in the mastectomy group (8.99%) compared to the BCT group (4.87%; *p* = 0.056). Estrogen Receptor (ER) positivity was higher in the BCT group (68.48%) compared to the mastectomy group (64.66%; *p* = 0.149). Similarly, Progesterone Receptor (PR) positivity was more common in the BCT group (59.53%) compared to the mastectomy group (57.41%; *p* = 0.326).

Table 3 presents the univariable and multivariable hazard ratios (HRs) for factors associated with survival. In the univariable analysis, patients who underwent breast-conserving therapy (BCT) demonstrated significantly better survival compared to those who underwent mastectomy, with a hazard ratio of 0.44 (95% CI, 0.29–0.67; *p* < 0.001). This association remained significant in the multivariable analysis, with an adjusted hazard ratio of 0.58 (95% CI, 0.36–0.93; *p* = 0.023). Age was significantly associated with survival in both univariable and multivariable analyses. Patients aged 50 years or older had a higher hazard ratio for mortality compared to those younger than 50 years in both univariable analysis (HR, 1.66; 95% CI, 1.11–2.47; *p* = 0.013) and multivariable analysis (HR, 1.66; 95% CI, 1.06–2.58; *p* = 0.025). Tumor size was significantly associated with survival in both analyses. Tumors measuring 2.1–5 cm had a higher hazard ratio compared to tumors measuring 0–2 cm in univariable analysis (HR, 1.53; 95% CI, 1.04–2.26; *p* = 0.031) and multivariable analysis (HR, 1.55; 95% CI, 1.00–2.41; *p* = 0.048). No significant associations were found between tumor grade and survival in either univariable or multivariable analysis. Nodal involvement showed borderline significance in univariable analysis, with N1 staging having an HR of 1.81 (95% CI, 1.01–3.24; *p* = 0.047). In the multivariable analysis, this association remained marginal, with an adjusted HR of 1.85 (95% CI, 0.99–3.46; *p* = 0.054). Molecular subtypes exhibited varied associations in univariable analysis. Luminal B tumors had a hazard ratio of 0.48 (95% CI, 0.24–0.96; *p* = 0.039), and HER2-enriched tumors had an HR of 0.69 (95% CI, 0.36–1.35; *p* = 0.282). However, these associations were not significant in the multivariable analysis, with adjusted HRs of 0.65 (95% CI, 0.29–1.46; *p* = 0.299) for Luminal B and 0.76 (95% CI, 0.36–1.59; *p* = 0.460) for HER2-enriched tumors.

Adjuvant chemotherapy showed no significant association with survival in either analysis. The hazard ratio was 1.22 (95% CI, 0.75–1.97; *p* = 0.417) in the univariable analysis and 1.03 (95% CI, 0.60–1.76; *p* = 0.921) in the multivariable analysis. Adjuvant hormonal therapy demonstrated significance in univariable analysis (HR, 0.66; 95% CI, 0.45–0.97; *p* = 0.037) but not in multivariable analysis (HR, 0.83; 95% CI, 0.43–1.60; *p* = 0.576). Adjuvant radiotherapy was not significantly associated with survival in univariable analysis (HR, 1.58; 95% CI, 0.93–2.70; *p* = 0.092) or multivariable analysis (HR, 1.53; 95% CI, 0.87–2.68; *p* = 0.138).

Table 4 presents survival rates at 5-year, 10-year, and 15-year intervals, stratified by breast cancer subtype and surgical treatment. The survival rates at 5, 10, and 15 years were evaluated across breast cancer subtypes, comparing outcomes between patients treated with breast-conserving therapy (BCT) and mastectomy. The overall survival rate at 5 years was 96.49% (95% CI, 93.36–98.16) for the BCT group and 88.64% (95% CI, 84.16–91.92) for the mastectomy group. At 10 years, survival rates decreased to 88.69% (95% CI, 83.61–92.27) for BCT and 74.09% (95% CI, 67.94–79.24) for mastectomy. By 15 years, survival rates further declined to 80.01% (95% CI, 70.78–86.60) for BCT and 64.33% (95% CI, 54.17–72.80) for mastectomy. Among patients with Luminal A breast cancer, the 10-year survival rates were 90.49% (95% CI, 82.32–94.99) and 81.05% (95% CI, 71.78–87.53) for BCT and mastectomy, respectively. By 15 years, survival rates declined to 80.19% (95% CI, 66.40–88.78) for BCT and 71.66% (95% CI, 58.06–81.53) for mastectomy.

For Luminal B patients, the 10-year survival rates were 94.30% (95% CI, 83.18–98.15) for BCT and 80.96% (95% CI, 64.85–90.21) for mastectomy. By 15 years, survival rates showed a marked decline to 58.38% (95% CI, 8.62–88.85) for BCT and 53.97% (95% CI, 9.93–84.82) for mastectomy.

Patients with HER2-enriched breast cancer had a 10-year survival rate of 92.44% (95% CI, 73.02–98.06) for BCT and 66.23% (95% CI, 49.73–78.43) for mastectomy. By 15 years, survival rates for BCT remained steady at 92.44% (95% CI, 73.02–98.06), while mastectomy survival rates further decreased to 59.61% (95% CI, 39.69–74.85).

For patients with triple-negative breast cancer, the 10-year survival rates were 78.13% (95% CI, 61.88–88.08) for BCT and 65.30% (95% CI, 47.78–78.20) for mastectomy. By 15 years, survival rates for both groups remained stable at 78.13% (95% CI, 61.88–88.08) for BCT and 65.30% (95% CI, 47.78–78.20) for mastectomy.

### 3.2. Survival Analysis

**Figure 1:**
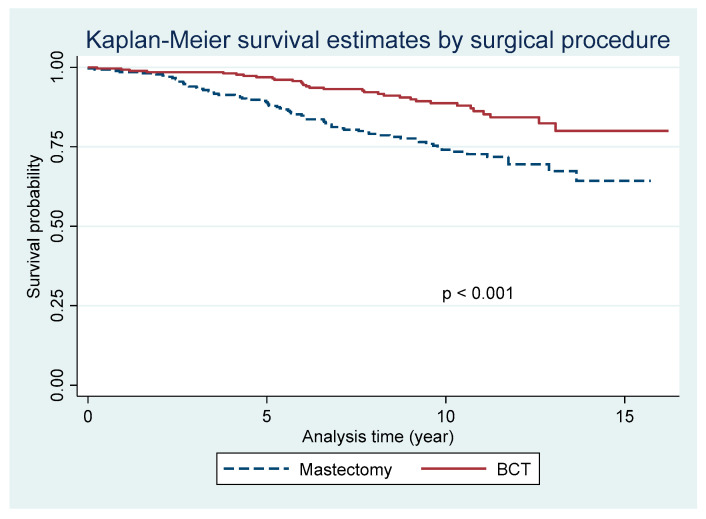
The Kaplan–Meier survival curve illustrates the overall survival probabilities for patients undergoing breast-conserving therapy (BCT) and mastectomy over a 15-year follow-up period.

Figure 1 The Kaplan–Meier survival analysis demonstrates a trend toward improved overall survival for patients undergoing breast-conserving therapy (BCT) compared to those treated with mastectomy. The survival probabilities were evaluated over a median follow-up time of up to 15 years. At 15 years, the survival probability for the BCT group was 80.01% (95% CI, 70.78 to 86.60), while the survival probability for the mastectomy group was 64.33% (95% CI, 54.17 to 72.80), and the difference reached statistical significance (*p* < 0.001).

**Figure 2:**
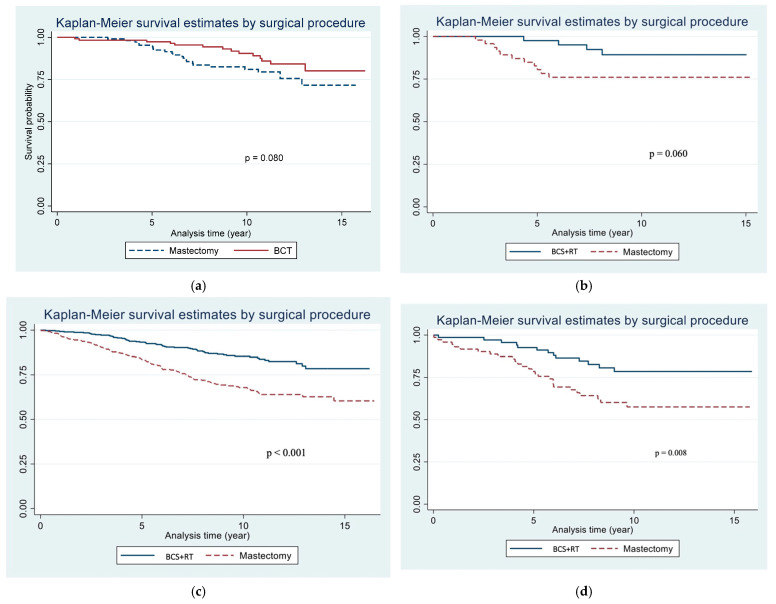
(**a**) Kaplan–Meier Survival Analysis of Overall Survival in the Luminal A Breast Cancer Subtype: Comparison of Breast-Conserving Therapy vs. Mastectomy. (**b**) Kaplan–Meier Survival Analysis of Overall Survival in the Luminal B Breast Cancer Subtype: Comparison of Breast-Conserving Therapy vs. Mastectomy. (**c**) Kaplan–Meier Survival Analysis of Overall Survival in the HER2-Enriched Breast Cancer Subtype: Comparison of Breast-Conserving Therapy vs. Mastectomy. (**d**) Kaplan–Meier Survival Analysis of Overall Survival in the Triple-Negative Breast Cancer Subtype: Comparison of Breast-Conserving Therapy vs. Mastectomy.

Figure 2 shows the comparison of overall survival by tumor subtype between the two surgical procedures. Specifically, Figure 2a shows that the Kaplan–Meier analysis demonstrates non-significantly different estimated overall survival at 15 years for the BCT group compared to the mastectomy group in the Luminal A breast cancer subtype, with overall survival rates of 80.19% (95% CI, 66.40 to 88.78) for the BCT group and 71.66% (95% CI, 58.06 to 81.53) for the mastectomy group (*p* = 0.08). Similarly, Figure 2b illustrates that the Kaplan–Meier analysis reveals non-significant difference of 15-year overall survival for the BCT group compared to the mastectomy group in the Luminal B breast cancer subtype, with survival rates of 58.38% (95% CI, 8.62 to 88.85) for the BCT group and 53.97% (95% CI, 9.93 to 84.82) for the mastectomy group (*p* = 0.06).

In contrast, Figure 2c presents a Kaplan–Meier analysis that demonstrates a significantly greater overall survival of the BCT group compared to the mastectomy group in HER2-enriched breast cancer. The 15-year overall survival rates were 92.44% (95% CI, 73.02 to 98.06) for the BCT group and 59.61% (95% CI, 39.69 to 74.85) for the mastectomy group (*p* < 0.001). Similarly, Figure 2d shows a Kaplan–Meier analysis that demonstrates a significant difference in overall survival between the two groups in the triple-negative breast cancer subtype. The 15-year overall survival rates were 78.13% (95% CI, 61.88 to 88.08) for the BCT group and 65.30% (95% CI, 47.78 to 78.20) for the mastectomy group (*p* = 0.008). This suggests a survival advantage favoring BCT over mastectomy in these two subtypes.

## 4. Discussion

For eight decades, breast-conserving therapy (BCT) has served as a standard treatment option for patients with early-stage breast cancer (EBC), presenting an alternative to mastectomy. A study analyzing the period from 1995 to 2002 [26,27,28,29] showed that clinical trials employing randomization demonstrated that mastectomy may be unnecessary for patients with stage I-II invasive breast cancer; instead, BCT or breast-conserving surgery (BCS) plus radiotherapy (RT) are equally effective concerning long-term survival outcomes. However, since 2013, a growing body of research has indicated that BCT may actually have higher survival rates than mastectomy for EBC [18,30,31,32].

Interestingly, a meta-analysis suggested that mastectomy provides a slight overall survival (OS) advantage when compared to BCT in the context of EBC, although this benefit is minimal and may not significantly impact clinical decision-making [23]. On the other hand, more recent meta-analysis studies have reported that BCT offers better OS rates than mastectomy [24,25]. This conflicting evidence highlights the ongoing debate regarding the optimal surgical approach for EBC treatment. As research continues to evolve, understanding these complexities will be essential for guiding clinical practice and improving patient care.

The primary outcome of our study demonstrates that BCT provides a significant survival advantage over mastectomy. The survival probability for the BCT group was 80.01% (95% CI, 70.78 to 86.60), compared to 64.33% (95% CI, 54.17 to 72.80) for the mastectomy group (*p* < 0.001) over a median follow-up period of up to 15 years. This supports findings from previous studies that highlight the potential benefits of breast-conserving therapy (BCT) over mastectomy in terms of overall survival (OS) [18,30,31,32]. Our study corroborates the increasing trend towards BCT, as seen in recent meta-analyses, which suggest that BCS, with the addition of radiotherapy, may offer superior long-term survival compared to mastectomy.

Interestingly, our study revealed that BCT offers a significant survival advantage over mastectomy in HER-2-enriched breast cancer, with a 15-year survival rate of 92.44% in the BCT group compared to 59.61% in the mastectomy group (*p* < 0.001). Additionally, this benefit extends to patients with triple-negative breast cancer (TNBC), where the BCT group demonstrated a 15-year survival rate of 78.13% compared to 65.30% in the mastectomy group (*p* = 0.008). Notably, this advantage was not observed in patients with Luminal EBC. This finding diverges from the existing literature, which suggests that hormone receptor-positive tumors respond well to conservative surgical approaches [33,34]. This is an important finding, as HER2-enriched and TNBC have historically been associated with a poorer prognosis.

Our findings demonstrated significant survival advantages of breast-conserving therapy (BCT) over mastectomy, particularly in HER2-enriched, as well as triple-negative subtypes. However, in HER2-positive patients, while trends favoring BCT were observed, this subgroup warrants further investigation to determine the true impact of surgical strategies on survival outcomes. We found limited access to anti-HER2 therapies, such as trastuzumab, during the study period. The treatment of breast cancer in Thailand reflects regional epidemiology, healthcare infrastructure, and resource availability while being broadly informed by international guidelines such as those from the National Comprehensive Cancer Network (NCCN) [35] and the European Society for Medical Oncology (ESMO) [36]. However, key differences exist. Screening in Thailand primarily relies on opportunistic approaches, emphasizing breast self-examinations and clinical breast exams, due to resource limitations and uneven access to mammography [37]. By contrast, Western guidelines advocate for routine population-based mammographic screening starting at ages 40 to 50 [38]. Treatment modalities in Thailand also diverge from those in Western settings. Thai patients more commonly undergo surgery, such as mastectomy or breast-conserving therapy, with limited access to adjuvant systemic therapies, which are often constrained by cost and availability [39]. Similarly, access to targeted therapies such as trastuzumab in Thailand remains limited due to economic barriers [40], a stark contrast to the widespread availability of biologics in Western practices.

The late introduction of trastuzumab into Thailand’s healthcare system and its restricted availability under national healthcare policies may have disproportionately influenced outcomes for HER2-positive patients [41], with systemic treatment disparities potentially playing a more decisive role than the choice of surgery. Future studies should aim to stratify HER2-positive patients based on access to targeted therapies, such as trastuzumab, to more precisely elucidate the contribution of surgical and radiotherapeutic approaches to survival outcomes. Our study acknowledges the potential confounding impact of systemic treatments, including chemotherapy and HER2-targeted therapies, on survival outcomes. During the study period, HER2-positive patients had limited access to trastuzumab, a factor that likely influenced our findings. Despite these limitations, HER2-positive patients treated with BCT demonstrated better survival outcomes compared to those undergoing mastectomy. These results suggest that localized treatment strategies, including surgery and radiotherapy, may provide inherent survival benefits independent of systemic therapy. Notably, some HER2-positive patients in our study achieved favorable outcomes even without receiving anti-HER2 therapy, highlighting the importance of localized treatment in enhancing survival. This underscores the complementary role of localized and systemic treatment modalities in managing HER2-positive breast cancer. Our findings suggest that while anti-HER2 agents play a critical role, their impact is not solely determinant, and a multimodal approach combining systemic and localized therapies may offer the most favorable outcomes.

Based on our study results, factors significantly associated with improved survival outcomes in EBC patients include the type of surgical procedure, with BCT showing favorable results, younger age at diagnosis, and tumor size. Specifically, tumors measuring 2 cm or smaller are associated with better survival compared to those larger than 2 cm. This suggests that even within EBC, smaller tumor sizes are indicative of more favorable survival outcomes.

Propensity score-matched analysis highlighted significant differences in patient demographics and tumor characteristics between the BCT and mastectomy groups. Patients undergoing BCT tended to be younger and had smaller tumors. These factors are the most likely to contribute to the improved survival outcomes observed in the BCT group.

Radiotherapy plays a crucial role in eliminating residual microscopic disease, thereby reducing the risk of local recurrence and improving long-term survival outcomes. However, our study’s multivariable analysis of survival-associated factors indicates that adjuvant radiotherapy is not significantly correlated with improved survival. Consequently, the survival advantages observed with BCT in EBC patients may not be attributable to the application of adjuvant radiotherapy. While radiotherapy does improve local control, this does not necessarily equate to an enhancement in overall survival, which can also be influenced by systemic therapies. Supporting this notion, a comprehensive meta-analysis by the Early Breast Cancer Trialists’ Collaborative Group (EBCTCG) involved data from 22 randomized trials comparing mastectomy with or without adjuvant radiation. It revealed a significant survival benefit primarily in patients with positive axillary lymph nodes, especially those with four or more positive nodes, tumors larger than 5 cm, or with skin invasion [42]. This underscores the benefit of radiotherapy in locally advanced cases rather than in typical EBC scenarios.

Our findings support the need for a more personalized approach to the surgical management of EBC. While mastectomy may still be appropriate for patients with larger tumors or extensive nodal involvement, BCT offers a viable alternative for many patients. The superior survival outcomes observed with BCT, especially in HER2-enriched and TNBC patients, suggest that breast conservation should be considered the first-line option in appropriately selected patients.

This study is characterized by limitations inherent to its single-institution, retrospective design. This design significantly narrows the diversity of patient demographics, clinical methodologies, and therapeutic protocols, consequently constraining the extrapolation of these results to a broader clinical setting. Moreover, variances in age and tumor dimensions between the groups undergoing breast-conserving therapy (BCT) and mastectomy may confound the survival outcomes observed. Predominantly, the superior overall survival noted in the BCT cohort could potentially be attributed to the younger patient age and smaller tumor size at diagnosis rather than the therapeutic modalities employed.

Furthermore, the limited use of anti-HER2 therapies, now considered standard in adjuvant treatment for HER2-positive patients, poses another limitation. Historically, the high cost of these therapies restricted access to only a small number of patients. Although access has improved following the inclusion of anti-HER2 therapies in public health services, the results from our subgroup analysis of HER2-enriched early breast cancer (EBC) patients who did not receive anti-HER2 treatment might not be generalizable to current populations who have access to these therapies.

These findings underscore the need for future research to adopt multi-institutional, prospective frameworks. Such studies should aim to encompass a more heterogeneous patient population, apply standardized investigative methodologies, and embrace a diversity of clinical practices. This approach would not only enhance the robustness and generalizability of the findings but also provide a more definitive comparison of the efficacy of BCT versus mastectomy across varied clinical landscapes.

## 5. Conclusions

This study demonstrates that breast-conserving therapy offers superior long-term survival compared to mastectomy in early-stage breast cancer patients, particularly among those with HER2-enriched and triple-negative subtypes. However, our study’s limitations may affect the results. Our findings emphasize the need for personalized treatment approaches to optimize outcomes based on tumor biology and patient characteristics.

## Figures and Tables

**Table 1:** Patient baseline characteristics before propensity score matching.

Parameters	BCT (*n* = 293)	Mastectomy (*n* = 1037)	*p*-Value
Age (Years), Mean (SD)	47.88 (10.17)	52.71 (10.70)	<0.001
Age (Years), *n* (%)			<0.001
<50	158 (59.18)	108 (40.45)	
≥50	109 (40.82)	159 (59.55)	
Marital status, *n* (%)			0.158
Married	237 (82.29)	878 (85.66)	
Single	51 (17.71)	147 (14.34)	
Menopausal status, *n* (%)			<0.001
No menopause	135 (54.88)	337 (37.91)	
Menopause	111 (45.12)	552 (62.09)	
Medical insurance, *n* (%)			0.001
UC	93 (32.29)	433 (42.08)	
SSS	31 (10.76)	89 (8.65)	
CSMBS	119 (41.32)	414 (40.23)	
Self-paid	45 (15.63)	93 (9.04)	
Tumor location, *n* (%)			0.001
Upper inner quadrant	66 (30.41)	150 (20.27)	
Lower inner quadrant	16 (7.37)	60 (8.11)	
Upper outer quadrant	99 (45.62)	327 (44.19)	
Lower outer quadrant	22 (10.14)	93 (12.57)	
Central quadrant	12 (5.53)	94 (12.70)	
Nipple	1 (0.46)	16 (2.16)	
Axillary tail	0 (0)	0 (0)	
others	1 (0.46)	0 (0)	
Tumor Size, Mean (SD), cm	1.73 (0.94)	2.16 (1.15)	<0.001
0–2 cm, *n* (%)	204 (69.62)	537 (51.78)	
2.1–5 cm, *n* (%)	89 (30.38)	500 (48.22)	
Nodal staging, *n* (%)			0.058
N0	279 (95.22)	952 (91.80)	
N1	14 (4.78)	85 (8.20)	
Histological type, *n* (%)			0.686
Ductal	270 (93.10)	967 (93.70)	
Non-ductal	20 (6.90)	65 (6.30)	
Tumor grade, *n* (%)			0.913
I	12 (4.65)	48 (5.05)	
II	147 (56.98)	553 (58.15)	
III	99 (38.37)	350 (36.80)	
Estrogen receptor (ER) status, *n* (%)			0.152
Negative	85 (30.69)	341 (35.41)	
Positive	192 (69.31)	622 (64.59)	
Progesterone receptor (PR) status, *n* (%)			0.370
Negative	111 (40.07)	416 (43.24)	
Positive	166 (59.93)	546 (56.76)	
HER2-status, *n* (%)			0.094
Negative	178 (64.49)	560 (58.70)	
Positive	98 (35.51)	394 (41.30)	
Molecular subtype, *n* (%)			0.047
Luminal A	19 (6.91)	96 (10.14)	
Luminal B	108 (39.27)	313 (33.05)	
HER2-enriched	98 (34.91)	394 (40.97)	
Triple negative	52 (18.91)	150 (15.84)	
Adjuvant chemotherapy, *n* (%)			1.000
No	69 (24.30)	248 (24.53)	
Yes	215 (75.70)	763 (75.47)	
Adjuvant hormonal therapy, *n* (%)			0.101
No	108 (38.71)	328 (33.30)	
Yes	171 (61.29)	657 (66.70)	
Adjuvant radiotherapy, *n* (%)			<0.001
No	58 (20.28)	652 (65.13)	
Yes	228 (79.72)	349 (34.87)	
Adjuvant HER2 targeted therapy, *n* (%)			0.767
No	266 (94.33)	949 (94.71)	
Yes	16 (5.67)	53 (5.29)	

Abbreviations: BCT, breast-conserving therapy; cm, centimeter; CSMBS, civil service medical benefits scheme; HER2, human epidermal growth factor receptor 2; *n*, number; SD, standard deviation; SSS, social security scheme; UC, universal coverage.

**Table 2:** Characteristics of patients after propensity score matching.

Parameters	BCT (*n* = 267)	Mastectomy (*n* = 267)	*p*-Value	Pre-Matched STD	Post-Matched STD
Age (Years), Mean (SD)	47.60 (10.14)	52.27 (10.78)	<0.001	0.463	0.445
Age (Years), *n* (%)					0.381
<50	158 (59.18)	108 (40.45)	<0.001	0.390	
≥50	109 (40.82)	159 (59.55)			
Tumor Size, *n* (%)					0.461
0–2	184 (68.91)	132 (49.44)	<0.001	0.371	
2.1–5	83 (31.09)	135 (50.56)			
Tumor grade, *n* (%)					−0.070
I	9 (3.83)	14 (5.62)	0.621	−0.035	
II	136 (57.87)	145 (58.23)			
III	90 (38.30)	90 (36.14)			
Nodal staging, *n* (%)					0.142
N0	254 (95.13)	235 (91.01)	0.056	0.139	
N1	13 (4.87)	32 (8.99)			
Estrogen receptor (ER), *n* (%)					−0.051
Negative	81 (31.52)	105 (35.34)	0.149	−0.100	
Positive	176 (68.48)	149 (64.66)			
Progesterone receptor (PR), *n* (%)					−0.067
Negative	104 (40.47)	128 (42.59)	0.326	−0.064	
Positive	153 (59.53)	125 (57.41)			
HER2-status, *n* (%)					0.075
Negative	166 (64.84)	153 (61.20)	0.408	0.119	
Positive	90 (35.16)	97 (38.80)			
Molecular subtype, *n* (%)					−0.039
Luminal A	17 (6.67)	31 (12.40)			
Luminal B	100 (39.22)	76 (30.40)	0.052	−0.038	
HER2-enriched	90 (34.51)	97 (38.40)			
Triple negative	50 (19.61)	47 (30.52)			

Abbreviations: BCT, breast-conserving therapy; cm, centimeter; HER2, human epidermal growth factor receptor 2; *n*, number; SD, standard deviation.

**Table 3:** Univariate and Multivariate analysis of factors associated with survival.

Parameters	Crude HR	95% CI	*p*-Value	Adjusted HR	95% CI	*p*-Value
Surgical procedure						
BCT	0.44	0.29–0.67	<0.001	0.58	0.36–0.93	0.023
Mastectomy	1.00	-	Reference	1.00	-	Reference
Age (Years)						
<50	1.00	-	Reference	1.00	-	Reference
≥50	1.66	1.11–2.47	0.013	1.66	1.06–2.58	0.025
Tumor size, cm						
0–2	1.00	-	Reference	1.00	-	Reference
2.1–5	1.53	1.04–2.26	0.031	1.55	1.00–2.41	0.048
Tumor grade				-		-
I	1.00	-	Reference			
II	1.13	0.41–3.11	0.819			
III	1.31	0.47–3.69	0.606			
Nodal staging						
N0	1.00	-	Reference	1.00	-	Reference
N1	1.81	1.01–3.24	0.047	1.85	0.99–3.46	0.054
Estrogen receptor (ER)						
Negative	1.00	-	Reference	1.00	-	Reference
Positive	0.65	1.00–1.02	0.038	1.03	0.50–2.14	0.932
Progesterone receptor (PR)						
Negative	1.00	-	Reference	1.00	-	Reference
Positive	0.64	0.42–0.96	0.030	0.94	0.48–1.82	0.853
HER2-status						
Negative	1.00	-	Reference	-	-	-
Positive	1.00	0.65–1.54	0.992			
Molecular subtype						
Luminal A	1.00	-	Reference	1.00	-	-
Luminal B	0.48	0.039	0.24–0.96	0.65	0.29–1.46	0.299
HER2-enriched	0.69	0.282	0.36–1.35	0.76	0.36–1.59	0.460
Triple negative	1.00	0.998	0.50–2.00	1.10	0.45–2.71	0.830
Adjuvant chemotherapy						
No	1.00	-	Reference	1.00	-	Reference
Yes	1.22	0.75–1.97	0.417	1.03	0.60–1.76	0.921
Adjuvant hormonal therapy						
No	1.00	-	Reference	1.00	-	Reference
Yes	0.66	0.45–0.97	0.037	0.83	0.43–1.60	0.576
Adjuvant radiotherapy						
No	1.00	-	Reference	1.00	-	Reference
Yes	1.58	0.93–2.70	0.092	1.53	0.87–2.68	0.138
Adjuvant HER2 targeted therapy						
No	1.00	-	Reference	1.00	-	Reference
Yes	1.19	0.58–2.45	0.639	1.13	0.51–2.52	0.765

Abbreviations: CI, confident interval; cm, centimeter; HER2, human epidermal growth factor receptor 2.

**Table 4:** Long-Term Survival Rates at 5, 10, and 15 Years by Breast Cancer Subtypes and Surgical Treatment.

Breast Cancer Subtype	Survival Rate (95% CI)					
	5-Year		10-Year		15-Year	
	BCT	Mastectomy	BCT	Mastectomy	BCT	Mastectomy
Total	96.49 (93.36–98.16)	88.64 (84.16–91.92)	88.69 (83.61–92.27)	74.09 (67.94–79.24)	80.01 (70.78–86.60)	64.33 (54.17–72.80)
Luminal A	97.38 (92.10–99.15)	94.35 (87.85–97.42)	90.49 (82.32–94.99)	81.05 (71.78–87.53)	80.19 (66.40–88.78)	71.66 (58.06–81.53)
Luminal B	98.33 (8875–99.76)	89.36 (76.31–95.43)	94.30 (83.18–98.15)	80.96 (64.85–90.21)	58.38 (8.62–88.85)	53.97 (9.93–84.82)
HER2-enrich	96.30 (76.49–99.47)	81.67 (67.72–90.01)	92.44 (73.02–98.06)	66.23 (49.73–78.43)	92.44 (73.02–98.06)	59.61 (39.69–74.85)
TNBC	93.47 (81.08–97.85)	82.16 (67.48–90.65)	78.13 (61.88–88.08)	65.30 (47.78–78.20)	78.13 (61.88–88.08)	65.30 (47.78–78.20)

Abbreviations: BCT, breast-conserving therapy; CI, confident interval; HER2, human epidermal growth factor receptor 2; TNBC, Triple-negative breast cancer.

## Data Availability

The data is unavailable due to privacy reasons. For further information, please contact the corresponding author.
